# Supplementary material for: The Molecular Characterization of a New Prunus-Infecting Cheravirus and Complete Genome Sequence of Stocky Prune Virus
Source: Viruses. 2022 Oct 23;14(11):2325. doi: 10.3390/v14112325 (PMC9698620; doi:10.3390/v14112325)
Supplement: Supplementary file 1 [file viruses-14-02325-s001.zip › viruses-1939373-supplementary.pdf]

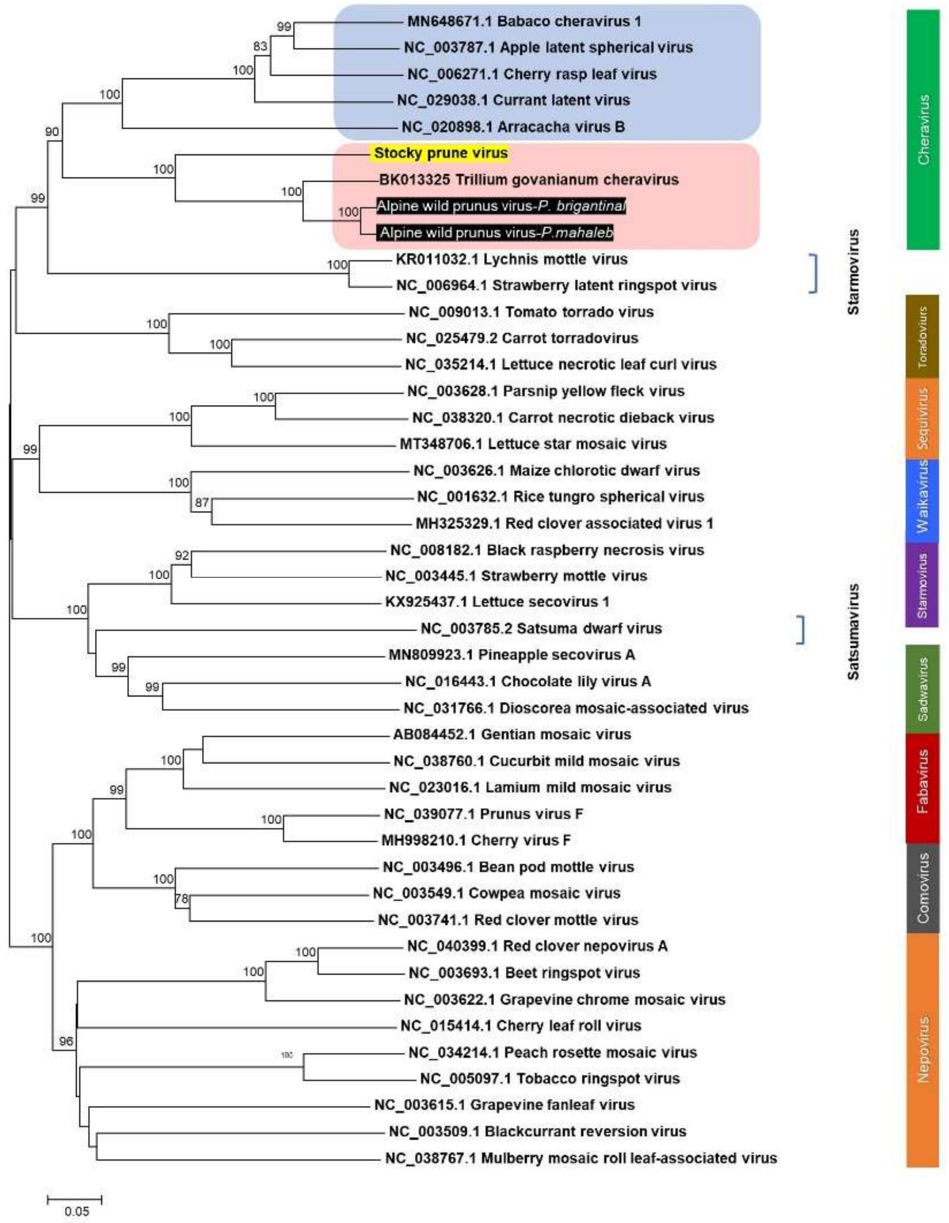

Figure S1. Phylogenetic tree reconstructed using the alignment of amino acid sequences of the Pro-Pol region (conserved domain between the "CG" motif of the 3C-protease and the "GDD" motif of the RdRp) as defined [28] of representative members of the family *Secoviridae*. The tree was generated using the neighbor joining method and strict aa identity p-distances. The statistical significance of branches was evaluated by bootstrap analysis (1,000 replicates). Bootstrap values less than 70% were removed. The scale bar represents 5% amino acid divergence. Accession numbers and names of the viruses included in the tree are indicated. Genera to which viruses belong are indicated at the right of the figure.

Table S1. List of primers used in this study, their purpose and their target molecule

Primer	Purpose	Target [1]	Sequence 5′-3′	combination
Pb-seco-GAP1F1	Gap filling	RNA1 AWPV-Pb	TCAGTAAGCGTGGACGTATA	Pb-seco-GAP1R1
Pb-seco-GAP1R1	Gap filling	RNA1 AWPV-Pb	CAATGGAAGTTGCGCAGCC	
Nseco-R1-5RACE-R1	5′ RACE	5'end RNA1 AWPV-Pb	GGAAGCCAGATGCCTCCGGTGGGA	Pb-seco-5F
Pb-seco-5F	5′ RACE	5'end RNA1 AWPV-Pb	GGGCAGAGCCTTCAGATACT	
Seco-PB-3RACE-R1	3′ RACE	3'end RNA1 AWPV-Pb	GCTGGATAGCCTGTGGCAAGTTCCTC	LD-prim
Nseco-R2-5RACE-4	5′ RACE	5'end RNA2 AWPV-Pb	ACTGGTGCAATGAGAGCAGAAGAGAGA	UPM
Seco-PB-3RACE-R2-2	3′ RACE	3'end RNA2 AWPV-Pb	CGGAACAATACAATCTCTTCAAG	LD-prim
Nseco-Pm-5RACE-R1	5′ RACE	5'end RNA1 AWPV-Pm	CTGCTCAGAGAGTGCCTCAATGGGCG	Pb-seco-5F
Nseco-Pm-3RACE-R1	3′ RACE	3'end RNA1 AWPV-Pm	AGACTGGAGCTTACCATACTGAACC	LD-prim
Nseco-Pm-5RACE-R2-2	5′ RACE	5'end RNA2 AWPV-Pm	AAGGCCCACATCTGCCAGGGACAAGGT	Pb-seco-5F
Nseco-Pm-3RACE-R2	3′ RACE	3'end RNA2 AWPV-Pm	CCTTGAGAGGTAGCGCAGCTATTGCAC	LD-prim
StPV-5RACE-RNA1	5′ RACE	5'end RNA1	GGCAGCACTTCCTCGGCTACGGGG	UPM
StPV-3RACE-RNA1	3′ RACE	3'end RNA1 StPV	TGGGGATGGCCTTAGCCAGGCTGT	LD-prim
StPV-5Race-RNA2-2	5′ RACE	5'end RNA2 StPV	CAGAGTACN$_5$GGTTTTCTTCTTGGCGTA	StPV-5RACE-RNA2
StPV-5RACE-RNA2	5′ RACE	5'end RNA2 StPV	GGGCGAGGTCGGTGCAGAGCTGAA	
StPV-3RACE-RNA2	3′ RACE	3'end RNA2 StPV	CGGGTCGGCCACAGAAACTTGGGGTAG	LD-prim
LD-prim	3′end	LD-PCR	CACTGGCGGCCGCTCGAGCATGTAC	
LD-poly T	3′end	cDNA synthesis	CACTGGCGGCCGCTCGAGCATGTACT$_{25}$N$_3$	
New-seco-CP-F	Detection	RNA2, AWPV-Pb, Pm	TTGATGGTCCCAGTATTGTTCC	New-seco-CP-R
New-seco-CP-R	Detection		TCCCATTTCCGTGCTTAAGAAT	
Seco-PA-R1-F	Detection	RNA1-AWPV-Pa	CTTTCTTGCGTACCTTGGCC	Seco-PA-R1-R
Seco-PA-R1-R	Detection		GAAGCTGCAGATTTCCCACA	
StPV RNA1 F1	Detection	RNA1-StPV	CTCTTGCAAATAAGGTTCGCA	StPV RNA1 R1
StPV RNA1 R1	Detection		GGGAGCTAAAACTCTACCAG	

[1] AWPV-Pb: Alpine wild prunus virus-*P. brigantina*; AWPV-Pm: Alpine wild prunus virus-*P. mahaleb;* AWPV-Pa: Alpine wild prunus virus-*P. armeniaca;* StPV: stocky prune virus
